# Gestational anemia and maternal antenatal and postpartum psychological distress in a prospective FinnBrain Birth Cohort Study

**DOI:** 10.1186/s12884-022-05032-z

**Published:** 2022-09-13

**Authors:** Lotta Kemppinen, Mirjami Mattila, Eeva Ekholm, Linda Huolila, Juho Pelto, Hasse Karlsson, Kaarin Mäkikallio, Linnea Karlsson

**Affiliations:** 1 Department of Obstetrics and Gynecology, Turku University Hospital, U-Hospital, Savitehtaankatu 5, 20520 Turku, Finland; 2 University of Turku, Turku, Finland; 3 Department of Clinical Medicine, University of Turku, Turku Brain and Mind Center, FinnBrain Birth Cohort Study, Turku, Finland; 4 Department of Psychiatry, University of Turku and Turku University Hospital, Turku, Finland; 5 Centre for Population Health Research, Turku University Hospital and University of Turku, Turku, Finland; 6 Department of Paediatrics and Adolescent Medicine, University of Turku and Turku University Hospital, Turku, Finland

**Keywords:** Antenatal depression, Anxiety, Depression, Gestational anemia, Iron deficiency anemia in pregnancy, Postpartum complication, Postpartum depression

## Abstract

**Background:**

Gestational anemia, most commonly caused by iron deficiency, may increase the risk of maternal anxiety and depression and have a potentially far-reaching impact on mother’s and newborn’s health. Several mechanisms, such as effects of iron deficiency on cerebral neurotransmitter metabolism, have been suggested. None of the earlier studies have assessed the association between gestational anemia and depression, anxiety and pregnancy-related anxiety simultaneously.

**Methods:**

Women, participating in the FinnBrain Birth Cohort Study and attending maternity welfare clinics in Turku, whose hemoglobin (Hb) values during pregnancy were available were included in this study (*n* = 1273). The study group consisted of 301 women with Hb levels < 11.0 g/dL at any time during pregnancy, and 972 women with Hb ≥ 11.0 g/dL were included in the control group. Symptoms of depression, anxiety, and pregnancy-related anxiety were assessed using the Edinburgh Postnatal Depression Scale (EPDS), Symptom Checklist-90 (SCL), and Pregnancy-Related Anxiety Questionnaire (PRAQ) questionnaires at 14, 24, and 34 gestational weeks, and EPDS and SCL were also performed 3 and 6 months postpartum.

**Results:**

Gestational anemia was not associated with an increased risk of depression either prenatally or postpartum when the analyses were adjusted for maternal age at birth, parity, smoking during pregnancy, maternal education, and gestational age. However, a weak connection was found between gestational anemia and prenatal anxiety in the early pregnancy. Furthermore, the analysis between women with Hb < 10.0 g/dL and those with Hb ≥ 10.0 g/dL showed an association between gestational anemia and anxiety in the late pregnancy, but otherwise no difference in psychological distress was found.

**Conclusions:**

No evidence supporting the association between gestational anemia and antenatal or postpartum depression was found. However, a weak connection between gestational anemia and antenatal anxiety was observed. This finding needs further investigation to establish timing and investigate causality.

## Background

In addition to somatic complications, anemia has been recognized as a potential risk factor for psychiatric symptoms, such as cognitive function disorders, depression and psychiatric disorders in various patient populations [1]. Several mechanisms, such as effects of iron deficiency on cerebral neurotransmitter metabolism, has been suggested to act as underlying mechanisms [2]. Gestational anemia, which is most commonly caused by iron deficiency, is known to affect approximately 40% of pregnant woman globally [3]. According to the WHO, gestational anemia is defined as a hemoglobin (Hb) level < 11.0 g/dL [4], and it is associated with prematurity [5–8], low birth weight [7–9] and Cesarean delivery [5, 7]. A few studies have reported a relationship between maternal anemia and postpartum depression [10, 11], but data on a possible association between gestational anemia and antenatal depression are scarce [12, 13]. Maternal anemia has been, however, identified as a potential risk factor for antenatal anxiety symptoms [14]. Depression and anxiety commonly affect the same patient population, since the comorbidity between the two is known to be high [15–17]. However, studies including the evaluation of both maternal depression and anxiety as regards to gestational anemia are missing.

The global prevalence of maternal antenatal and postpartum depression is estimated to be around 15% and 17%, respectively, but the percentages vary by the definition of depression and the assessment method [18, 19]. Known risk factors of postpartum depression include psychosocial factors, such as prior history of depression and fear of childbirth [20], socioeconomical factors, such as adolescent or advanced maternal age and low socioeconomic status [20] and physiological factors, such as gestational diabetes and pre-eclampsia [21, 22]. Antenatal depression is most prevalent in the last trimester of pregnancy, and it is associated with poor nutrition, smoking, and alcohol consumption during pregnancy, which all pose a risk for the developing child, whereas postpartum depression is known to impair mother–newborn bonding [18]. Various mechanisms, such as iron status in the brain and its effect on neurotransmitter metabolism, postpartum hemorrhage, and impaired breastfeeding, have been proposed to explain the increased risk of depression in anemic patients [23]. Furthermore, iron supplementation given to non-anemic mothers with postpartum depression has been shown to significantly improve depressive symptoms [24].

Although several studies and a recent meta-analysis have recognized gestational anemia as a potential risk factor for maternal depression, the data are controversial, especially regarding the role of gestational anemia in antenatal depression. Recently, Maeda et al. [25] found no association between antenatal anemia and postpartum depression although a previous meta-analysis reported a link between antenatal anemia and postpartum depression [7]. A significant association between postpartum anemia and postpartum depression have been reported in several studies [23–25]. Thus, the data regarding an association between gestational anemia and antenatal and postpartum maternal depression remains inconclusive.

Pregnancy-related anxiety is a distinct form of anxiety that may be experienced by women during pregnancy and is typically normally distributed in the general population [26]. It is characterized by fears and worries that are specific to pregnancy (e.g. fear of giving birth or worries on child’s wellbeing). Changes in pregnancy-related anxiety have been linked to changes in several variables used to evaluate cognitive development in offspring [27]. The association is thought to be explained by changes in maternal physiology [28]. The role of maternal anemia in pregnancy-related anxiety is unknown.

In the present study, we hypothesized that pregnant women suffering from gestational anemia are at a greater risk for psychological distress during both pregnancy and early postpartum period. Specifically, we wanted to evaluate whether gestational anemia plays a role in the development of antenatal and postpartum depression and anxiety symptoms. Further we wanted to examine whether gestational anemia is related to pregnancy-related anxiety, as this has not been investigated by the earlier literature. By depicting the potential influence of gestational anemia on differential domains of maternal psychological wellbeing, we aim at improving the possibilities for developing holistic supportive and treatment strategies.

## Methods

The FinnBrain Birth Cohort study [29] consisted of 3808 women recruited in maternal prenatal care clinics in the Hospital District of Southwest Finland and Åland Islands, Finland, between December 2011 and April 2015. For participation, a verified pregnancy and sufficient knowledge of one of the official languages in Finland, Finnish, or Swedish, were required. Pregnant women, who attended maternity welfare clinics in Turku and whose Hb values during pregnancy were available (*n* = 1273) were included in the present study. Approval was obtained from the local ethics committee (57/1801/2011). A written informed consent was required at study entry.

In Finland, national guidelines suggest Hb measurement in each trimester of pregnancy. Hb concentrations, detected from intravenous blood samples, were collected from patient records from all study subjects in three periods: 20 weeks, 20–30 weeks, and > 30 weeks. In the present study, pregnant women with antenatal Hb level below 11.0 g/dL at any of the three measurement points were assigned to the anemic study group.

Maternal psychological distress was assessed using internationally validated, self-reported questionnaires, which pregnant women filled in online or sent via postal mail. Depressive symptoms were assessed using the Edinburgh Postnatal Depression Scale (EPDS), which has been validated in several countries [30, 31]. In Finland, EPDS is in a routine clinical use for screening maternal antenatal and postpartum depression, and with its ten questions on a 4-point Likert scale from 0 to 3, the total score varies between 0–30. EPDS ≥ 10 indicates depressive symptoms [32]. Symptom Checklist-90 (SCL) was used to evaluate general anxiety. It consists of ten questions rated from 0 to 5, with the total score ranging between 0–50. SCL has been proven to be reliable for assessing anxiety symptoms in research and clinical settings [33, 34]. To evaluate maternal stress and adaptation to pregnancy we used PRAQ-R2 (Pregnancy-Related Anxiety Questionnaire-Revised), a revised version of the PRAQ, which is suitable for primiparous and multiparous women, to estimate pregnancy-related anxiety typical for this major transitional period in one’s life [35, 36]. PRAQ-R2 consists of 10 questions scored from 1 to 5; the total score ranges thus between 5–50. All three questionnaires, EPDS, SCL, and PRAQ, were assessed at 14, 24, and 34 gestational weeks, depicting the three trimesters of pregnancy, each of which have different physical, mental, developmental, and maturational goals. EPDS and SCL were also evaluated at 3 and 6 months postpartum. The association between gestational anemia (Hb < 11.0 g/dL) and psychological distress was assessed using EPDS, SCL, and PRAQ.

Information on parity (0 = nulliparous, 1 = multiparous), maternal BMI (kg/m2), gestational age (weeks), threating prematurity (0 = no, 1 = yes), antenatal corticosteroids (0 = no, 1 = yes), duration of labor (minutes), induction of labor (0 = no, 1 = yes), mode of delivery (1 = vaginal, 2 = cesarean section), red blood cell transfusion (0 = no, 1 = yes), gestational age at delivery (weeks), birth weight (grams), sex of newborn (0 = female, 1 = male), umbilical artery (pH), umbilical vein (pH), neonatal intensive care (0 = no, 1 = yes) were obtained from the Medical Birth Register kept by the National Institute for Health and Welfare (www.thl.fi). Maternal self-report was used to collect data on hypertension (0 = no, 1 = yes), type 1 diabetes (0 = no, 1 = yes), type 2 diabetes (0 = no, 1 = yes) and use of SSRI (selective serotonin reuptake inhibitor) /SNRI (selective norepinephrine reuptake inhibitor) medication (0 = no, 1 = yes), alcohol consumption (0 = no, 1 = any alcohol consumption), smoking during pregnancy (0 = no smoking, 1 = any smoking) and the level of education (1 = secondary school, 2 = high school or vocational education, 3 = university or polytechnic degree or higher).

### Statistical analysis

The statistical analyses were conducted using SPSS software (IBM® SPSS Statistics 25.0). Chi-square test and Fisher’s exact test were used to detect possible categorical differences in maternal and neonatal parameters between the studied groups. The distribution of the data was evaluated using visual assessment and the Kolmogorov–Smirnov and Shapiro-Wilks tests. The independent sample t-test was employed if the data were normally distributed. Otherwise, the Mann–Whitney U- test was chosen. General linear model univariate analysis was used to assess the relationship between maternal anemia and EPDS, SCL, and PRAQ at three different pregnancy time points and postpartum. Univariate analysis was adjusted for known risk factors for gestational anemia: maternal age [5], smoking during pregnancy [37], parity [38], maternal education [5], and gestational age [5]. Logistic regression was used to evaluate the difference in the incidence of high EPDS values (≥ 10) between the studied groups and the analysis was adjusted for the same risk factors. A *p*-value of < 0.05 was considered statistically significant.

## Results

Of the 3808 women recruited in the FinnBrain study, 1273 women from Turku with available Hb data were included in the current study, and 301 (23.6%) of this cohort had an antenatal Hb level < 11.0 g/dL at least in one of the measurement points at < 20 weeks, 20–30 weeks, or > 30 weeks during pregnancy. Pregnant women whose Hb levels were ≥ 11.0 g/dL in all three measurement points served as controls (*n* = 972). Maternal and neonatal characteristics of the studied groups are presented in Table 1. The anemic women had lower pre-pregnancy BMI compared to the controls (22.0 vs. 23.5, *p* < 0.001). Smoking during the late pregnancy and multiparity were also more common in the anemia group. Neonatal outcome was similar in both groups.

**Table 1:** Maternal characteristics and outcomes in pregnancies with either maternal hemoglobin (Hb) < 110 g/l or Hb ≥ 110 g/l. The values given are median (range) or n (%)

	**Hb < 110 g/l** **during pregnancy** ***n* = 301**	**Hb ≥ 110 g/l** **during pregnancy** ***n* = 972**	***p*-value**
**Maternal factors**			
Age at delivery (years, median(range))	30 (18–45)	30 (17–44)	0.658
Primiparity, n(%)	155 (52.5%)	612 (64.2%)	< 0.001*
Body mass index median(range)	22 (16–45)	23.5 (17–46)	< 0.001*
Prior Cesarean delivery, n(%)	21 (7.1%)	55 (5.8%)	0.398
Pre-pregnancy hypertension*, n(%)	4 (1.8%)	18 (2.3%)	0.799
Type I diabetes, n(%)	1 (0.4%)	0	
Type II diabetes, n(%)	2 (0.9%)	2 (0.3%)	
SSRI/SNRI medication during pregnancy, n(%)			
*I trimester*	7 (3.1%)	24 (3.0%)	0.985
*III trimester*	5 (2.6%)	18 (2.6%)	1.000
Smoking, n(%)			0.016*
*I trimester*	31 (10.4%)	99 (10.2%)	
*III trimester*	30 (10.0%)	55 (5.7%)	
Alcohol consumption, n(%)			
*I trimester*	46 (20.4%)	192 (24.6%)	0.213
*III trimester*	22 (11.6%)	75 (10.8%)	0.696
Level of education, n(%)			0.579
*Secondary school*	83 (36.2%)	267 (33.4%)	
*High school or vocational education*	65 (28.4%)	220 (27.5%)	
*University or polytechnic degree or higher*	81 (35.4%)	312 (39.0%)	
**Pregnancy**			
Hb < 20 weeks, median(range)	122 (86–148)	130 (111–153)	< 0.001*
Hb 20–30 weeks, median(range)	108 (91–128)	121 (110–143)	< 0.001*
Hb > 30 weeks, median(range)	108 (87–132)	124 (110–149)	< 0.001*
Threatening prematurity, n(%)	2 (0.7%)	2 (0.2%)	0.239
Antenatal corticosteroids, n(%)	11 (3.7%)	18 (1.9%)	0.067
**Delivery and postpartum period**			
Duration of labor (minutes, median(range))	430 (38–2066)	464 (39–2204)	0.196
Induction of labor, n(%)	70 (23.7%)	233 (24.5%)	0.794
Mode of delivery, n(%)			
Vaginal delivery	242 (82.0%)	778 (81.6%)	0.877
Cesarean section	53 (18.0%)	175 (18.4%)	
Cesarean section urgency			0.931
*Elective*	20 (6.8%)	62 (6.5%)	
*Emergency*	30 (10.2%)	101 (10.6%)	
*Crash emergency*	3 (1.0%)	12 (1.3%)	
Red blood cell transfusion, n(%)	14 (4.7%)	25 (2.6%)	0.083
**Neonatal outcome**			
Gestational age at delivery, median(range)	39.9 (33.2–42.2)	40.1 (29.2–42.4)	0.073
Birth weight (g, median(range))	3634 (1560–4950)	3550 (820–5200)	0.512
Male, n(%)	147 (49.2%)	500 (51.8%)	0.423
Umbilical artery pH, median(range)	7.28 (6.99–7.48)	7.27 (6.80–7.54)	0.373
Umbilical vein pH, median(range)	7.36 (7.11–7.52)	7.35 (6.94–7.52)	0.387
Neonatal intensive care, n(%)	53 (18.0%)	128 (13.4%)	0.053

^*^Self-reported diagnosis

As shown in Table 2, EPDS medians of the total sum scores measured during pregnancy did not differ statistically between the women with and without gestational anemia. The median (minimum – maximum) SCL score in the early pregnancy was 3.0 (0–30) in the anemic women and 2.0 (0–28) among controls (difference between the groups *p* = 0.043), and in the late pregnancy the respective values were 2.0 (0–25) and 2.0 (0–27), *p* = 0.756. Further, we found no evidence that antenatal PRAQ scores would differ by the anemia status of the mother during pregnancy except the score being one point higher in the women with Hb ≥ 11 g/dL (*p* = 0.029) in mid-pregnancy. In the late pregnancy, 14.8% of the anemic women had EPDS sum score ≥ 10, indicating possible depression, and the respective number in the control group was 13.6%. No significant differences in postpartum EPDS and SCL scores, assessed 3 and 6 months after delivery, were detected between women with and without anemia during pregnancy.

**Table 2:** Antenatal and postpartum depressive symptoms, and general anxiety and pregnancy related anxiety symptoms in women with and without gestational anemia. The values given are unadjusted median (minimum – maximum) or n (%) in each group, the estimated difference of the mean scores (B) or the adjusted odds ratio (OR) for the risk of postpartum depression between the anemic and non-anemic women from a linear or logistic regression models

	**Hb < 110 g/l** **during pregnancy** ***N* = 301**	**Hb ≥ 110 g/l** **during pregnancy** ***N* = 972**	**B/OR**^**a**^	**CI 95%**	***p*-value**
**Antenatal depressive symptoms**					
EPDS Early pregnancy	5.0 (0–26)	5.0 (0–27)	0.24	-0.37–0.85	0.446
EPDS Mid-pregnancy	4.0 (0–22)	4.0 (0–25)	-0.11	-0.73–0.52	0.740
EPDS Late pregnancy	4.0 (0–19)	4.0 (0–19)	0.15	-0.49–0.79	0.642
EPDS ≥ 10 Late pregnancy	28/189 (14.8%)	95 (13.6%)	OR 0.96	0.59–1.57	0.882
**Postpartum depressive symptoms**					
EPDS 3 months postpartum	4.0 (0–17)	3.0 (0–21)	0.30	-0.38–0.98	0.381
EPDS 6 months postpartum	3.0 (0–24)	3.0 (0–22)	0.11	-0.72–0.94	0.795
EPDS ≥ 10 3 months postpartum	16/167 (9.6%)	60 (9.9%)	OR 1.01	0.54–1.85	0.988
EPDS ≥ 10 6 months postpartum	20/139 (14.4%)	55 (10.7%)	OR 1.32	0.75–2.31	0.334
**Antenatal general and pregnancy-related anxiety symptoms**					
SCL Early pregnancy	3.0 (0–30)	2.0 (0–28)	0.62	0.02–1.23	0.043*
SCL Mid-pregnancy	3.0 (0–21)	3.0 (0–29)	0.19	-0.47–0.85	0.575
SCL Late pregnancy	2.0 (0–25)	2.0 (0–27)	0.10	-0.55–0.76	0.756
PRAQ Early pregnancy	21.0 (12–40)	22.0 (11–45)	-0.10	-2.45–2.25	0.933
PRAQ Mid-pregnancy	22.0 (10–43)	23.0 (10–46)	-1.17	-2.22– (-0.12)	0.029*
PRAQ Late pregnancy	24.0 (10–50)	23.0 (10–49)	-0.35	-1.45–0.75	0.535
**Postnatal anxiety symptoms**					
SCL 3 months postpartum	1.0 (0–17)	1.0 (0–24)	0.23	-0.40–0.86	0.469
SCL 6 months postpartum	1.0 (0–20)	1.0 (0–29)	0.16	-0.63–0.94	0.693

*EPDS* Edinburgh Postnatal Depression Scale, *SCL* Symptom Checklist-90 score, *PRAQ* (Pregnancy-Related Anxiety Questionnaire score

^a^Adjusted for maternal age, parity and smoking during pregnancy, maternal education, gestational age

To explore whether the severity of gestational anemia impacts maternal psychological distress, we performed an analysis comparing the outcomes of women with at least one prenatal Hb < 10.0 g/dL (*n* = 39) and women with Hb ≥ 10.0 g/dL (*n* = 1182) at all three measurement points. Pregnant women with Hb < 10.0 g/dL were younger 28 (18–45) years vs. 30 (17–45) years, (*p* = 0.024), and more frequently multiparous (56.4% vs. 37.3%, *p* = 0.019) than the control group, and smoking during the late pregnancy was also more common in this group (17.9% vs. 5.8%, *p* = 0.008). Women in the control group had higher educational level than women with an Hb value < 10.0 g/dL (*p* = 0.042). Gestational age at delivery was lower in women with Hb < 10.0 g/dL than in the controls (39.7 weeks vs. 40.1 weeks, *p* = 0.032), but the difference was not considered clinically significant. Table 3 shows that Hb < 10.0 g/dL during pregnancy was not associated with EPDS scores. In the antenatal anxiety symptoms assessment, the SCL score measured in the late pregnancy of pregnancy was higher in the group with Hb < 10.0 g/dL than those with Hb ≥ 10.0 g/dL (5.5 (0–14.0) vs. 2.0 (0–28.0), *p* = 0.028), but the SCL scores in the early and mid-pregnancy as well as during the postpartum period did not differ between the groups. Neither did we detect any differences between these groups regarding maternal PRAQ scores.

**Table 3:** Antenatal and postpartum depressive symptoms, and general anxiety and pregnancy related anxiety symptoms in women with and without severe gestational anemia. The values given are unadjusted median (minimum – maximum) or n (%) in each group, the estimated difference of the mean scores (B) or the adjusted odds ratio (OR) for the risk of postpartum depression between the anemic and non-anemic women from a linear or logistic regression models

	**Hb < 100 g/l** **during pregnancy** ***N* = 39**	**Hb ≥ 100 g/l** **during pregnancy** ***N* = 1182**	**B/OR**^**a**^	**CI 95%**	***p*-value**
**Antenatal depressive symptoms**					
EPDS Early pregnancy	5.0 (2–18)	5.0 (0–27)	0.99	-0.62–2.60	0.226
EPDS Mid-pregnancy	6.5 (1–20)	4.0 (0–25)	1.63	-0.08–3.33	0.062
EPDS Late pregnancy	5.8 (2–15)	4.0 (0–19)	1.70	-0.14–3.54	0.070
EPDS ≥ 10 Late pregnancy	5/18 (27.8%)	112 (13.2%)	OR 1.78	0.55–5.81	0.336
**Postpartum depressive symptoms**					
EPDS 3 months postpartum	5.0 (0–10)	3.0 (0–21)	1.22	-0.57–3.00	0.182
EPDS 6 months postpartum	4.0 (0–14)	3.0 (0–23)	0.38	-1.67–2.43	0.715
EPDS ≥ 10 3 months postpartum	3/19 (15.8%)	69 (9.5%)	OR 1.90	0.52–6.94	0.328
EPDS ≥ 10 6 months postpartum	2/17 (11.8%)	69 (11.2%)	OR 0.78	0.17–3.58	0.748
**Antenatal general and pregnancy-related anxiety symptoms**					
SCL Early pregnancy	3.0 (0–14)	2.0 (0–28)	0.77	-0.81–2.35	0.339
SCL Mid-pregnancy	4.5 (0–15)	3.0 (0–29)	1.64	-0.15–3.43	0.073
SCL Late pregnancy	5.5 (0–14)	2.0 (0–27)	2.10	0.22–3.98	0.028*
PRAQ Early pregnancy	24.5 (11–40)	22.0 (11–45)	1.73	-10.65–14.10	0.783
PRAQ Mid-pregnancy	27.0 (12–34)	23.0 (10–46)	1.89	-0.97–4.76	0.195
PRAQ Late pregnancy	-	23.0 (10–50)			
**Postnatal anxiety symptoms**					
SCL 3 months postpartum	2.0 (0–9)	1.0 (0–24)	0.97	-0.69–2.64	0.252
SCL 6 months postpartum	2.0 (0–13)	1.0 (0–29)	1.24	-0.72–3.19	0.214

*EPDS* Edinburgh Postnatal Depression Scale, *SCL* Symptom Checklist-90 score, *PRAQ* (Pregnancy-Related Anxiety Questionnaire score

^a^Adjusted for maternal age, smoking during pregnancy, parity, maternal education, and gestational age

## Discussion

According to the present study conducted in a general population-based pregnancy cohort, we found weak connections between anemia and anxiety. However maternal gestational anemia was not associated with maternal depressive symptoms either during pregnancy or the first six months of the postpartum period.

Most studies assessing the relationship between antenatal and postpartum anemia and postpartum depression have used EPDS in the evaluation of depression symptoms. In the meta-analysis by Azami et al., the number of anemic women ranged between 12–181 in eight of the included studies, with over 50% of the studies having a sample size less than 100 anemic women. According to their analyses, anemia during pregnancy was associated (RR = 1.240 (95% CI: 1.001–1.536, *p* = 0.048)) with postpartum depression risk at 24 h–6 weeks postpartum [10]. In a recent large meta-analysis, Kang et al. reported that maternal anemia increased the risk of having depression symptoms and/or depression diagnoses both antenatally (OR/RR = 1.36, 95% CI: 1.07–1.72) and postpartum (OR/RR = 1.53, 95% CI: 1.32–1.78)) [23]. However, contradictory reports have also been published [25, 39]. According to a prospective study by Maeda et al. [25], no associations between anemia during second and third trimester, and antenatal and postpartum depression were detected among 977 women, 197–435 of whom had Hb < 10.0–11.0 g/dL in the two last trimesters of pregnancy. This is in line with our findings with a special focus on the late pregnancy and the postpartum period. In our cohort, the incidence of a high EPDS score (EPDS ≥ 10) in the late pregnancy was 14.8% in the study group and 13.6% among the controls, which corresponds to prevalence rates in previously published reports in general population based samples [18]. Postpartum hemorrhage has been suggested to act as a mediator between anemia and depressive symptoms. Maeda et al. [25] proposed that postpartum hemorrhage and consequent anemia affects maternal experience of delivery, and therefore predisposes to depression. In our cohort, red blood cell transfusion rates did not differ between the studied groups, indicating no significant differences in the rates of significant postpartum hemorrhages between the groups. This may partly explain why anemia was not associated with postpartum depression in our study. Moreover, the multifactorial origin of depression in general contributes to the fact that potential specific association between gestational anemia and depression would be weak, especially a non-clinical population.

We found weak associations between gestational anemia and general anxiety symptoms between SCL in the early pregnancy and Hb < 11.0 g/dL and especially with SCL in the late pregnancy and Hb < 10.0 g/dL. The link between anemia and anxiety has been established in previous studies [1, 40]. One of the possible mechanisms is the effect of iron deficiency on brain dopaminergic and other monoamine metabolism [2, 41]. Furthermore, the PRAQ score in mid-pregnancy was one point higher in women with Hb ≥ 110 g/dL compared to women with anemia. Albeit statistically significant, this one point difference in the median total scores between the groups in one measurement is small from a clinical perspective, and the scores had roughly similar ranges in both groups. Thus, we conclude that this observed inverse association needs to be replicated and investigated further to gain more understanding on its relevance. Extremes of pregnancy-anxiety may reflect aberrant reactions to pregnancy or problems in adjusting to the life changes but also relevant reactions to pregnancy complications. Pregnancy-related anxiety has been linked with a range of offspring developmental outcomes [27, 42, 43], which underlines the importance of understanding its physiological as well as psychological underpinnings. PRAQ seems to measure a phenomenon different from general anxiety [26], but we are not aware of earlier reports assessing PRAQ, SCL and EPDS simultaneously in the context of gestational anemia.

SCL and EPDS symptom levels seemed to be lower during the early postpartum period than during pregnancy both in anemic and non-anemic women. This finding is in line with earlier research suggesting that there is a tendency for anxiety and depressive symptoms to drop during the very first months of postpartum [44, 45]. This decline may be due behavioral reasons (e.g. relief over uncomplicated delivery [44]) or beneficial hormonal changes during the initial transitions from pregnancy to postpartum period [46]. Taken together, it seems that gestational anemia does not influence this phenomenon.

The strengths of our study are its prospective study setting as well as assessing depressive, general anxiety, and pregnancy-related anxiety symptoms simultaneously. In most previous studies, EPDS has been used as a single tool to assess maternal psychologic distress. Getting similar results with three different symptom measures across pregnancy trimesters in the current study enhances the significance of our findings as well as the sample size of this study. Some limitations of our study are acknowledged. The study setting allowed for assessment of the association between gestational anemia and depressive and anxiety symptoms, however no conclusions of the causality and timing can be drawn due the data collection procedure that combined data from regular health care visits and research protocol. Symptoms were assessed using self-report questionnaires, which might overestimate the prevalence of depressive symptoms. However, overestimation is likely to be similar in both groups, and the frequency of depressive symptoms is in line with previous general population based studies [47]. Since anemia and depression cause partially similar symptoms, it could be argued that overestimation of depressive symptoms might have been greater among anemic women. This underlines further our non-associative results between gestational anemia and maternal depressive symptoms. Furthermore, several measurement points during pregnancy and postpartum allowed the assessment of changes in depression and anxiety symptoms, thus increasing the reliability of our findings although we acknowledge that all women were advised to initiate oral iron substitution when gestational anemia was detected.

## Conclusions

In our prospectively collected general population-based cohort, gestational anemia did not associate with maternal depressive symptoms. In turn, general anxiety symptoms during pregnancy were related to anemia status and deserves further research as maternal prenatal anxiety symptoms are important for both maternal and offspring well-being as such. We found no evidence on gestational anemia associating with postpartum depressive and anxiety symptoms.

## Data Availability

Finnish federal legislation on personal data protection in medical research precludes open sharing of data. Collaboration inquiries can be directed to the PIs of the FinnBrain Birth Cohort Study (hasse.karlsson@utu.fi AND linnea.karlsson@utu.fi).

## Abbreviations

Hb: Hemoglobin
EPDS: Edinburgh Postnatal Depression Scale
SCL: Symptom Checklist-90
PRAQ-R2: Pregnancy-Related Anxiety Questionnaire-Revised
PRAQ: Pregnancy-Related Anxiety Questionnaire
BMI: Body Mass Index
